# Investigating self-reported health by occupational group after a 10-year lag: results from the total Belgian workforce

**DOI:** 10.1186/s13690-018-0313-1

**Published:** 2018-11-08

**Authors:** Laura Van den Borre, Patrick Deboosere

**Affiliations:** Interface Demography, Department of Sociology, Vrije Universiteit Brussel, Pleinlaan 2, 1050 Brussels, Belgium

**Keywords:** Cohort study, Occupational health, Self-rated health, Men, Women, Occupation, Health inequalities

## Abstract

**Background:**

Belgium lacks a systematic overview of health differences by occupation. This is the first study to examine self-reported health among 27 occupational groups in Belgium with a lag time of 10 years.

**Methods:**

Individual data are derived from an anonymous linkage between the 1991 and 2001 Belgian census. The total working population (25–55 years) is selected from the 1991 Belgian census. Self-reported health (1 = fair or (very) bad health; 0 = (very) good health) was obtained from the 2001 census. Logistic regression analysis was used to analyse the health of 1.5 million men and 1.0 million women by occupational group in 1991. The active sex-specific population in 1991 and 2001 was the reference group. Controls include age, activity status and housing status at the time of 2001 census.

**Results:**

Both male and female workers in physically demanding occupations were more likely to report poor health. The three occupations with the highest age-adjusted Odds Ratios (OR) were extraction and building trade workers (OR_male_ 2.08 95% Confidence Interval (CI) 2.05–2.10; OR_female_ 2.15 CI 1.93–2.40); services elementary workers (OR_male_ 2.06 CI 2.03–2.10; OR_female_ 2.37 CI 2.34–2.41); and labourers in construction, manufacturing and transport (OR_male_ 1.90 CI 1.86–1.93; OR_female_ 2.21 CI 2.12–2.29). Men and women in teaching, scientific, health-related and managerial positions had the lowest age-adjusted ORs for poor self-reported health. The pattern in occupational health differences remained the same after controlling for activity status and socio-economic position.

**Conclusions:**

Occupational health inequalities are apparent after a lag time of 10 years. The identification of types of workers in poor health provide valuable insights to future health promotion strategies in the Belgian workforce.

## Background

Belgium has no systematic overview of occupational health differences. Yet, this issue is becoming increasingly important as policy measures are being developed to encourage workers to stay employed longer. Considering the importance of deteriorating health as a motive to leave employment [1], there is a high need to understand health inequalities in the Belgian workforce.

The available insights have been gained largely from international research. Manual work has been associated with poor health [2–4]. Although manual workers generally have a better health at the start of employment, their health declines more rapidly during working years than non-manual workers’ health [2, 4]. Longitudinal studies show that work-related health differences persist even after job changes or retirement [5]. Not only do manual workers have more years in poor health, they also have shorter life expectancies [6, 7]. Differences are explained partly by the physical demands of manual labour.

Research has been conducted on occupational health in Belgium, but the focus laid mainly on specific work-related diseases [8, 9], specific work contexts [10, 11] or mechanisms of health differences [12, 13]. Very little is known about health in specific occupations or how occupational health differences relate to each other. Which occupations have the best health situation? Which workers experience the most health problems? How large is occupational variation in health among Belgian workers?

This study follows the total Belgian workforce of 1991 using newly available census-linked data to investigate variations in self-reported health for specific occupational groups after a lag time of 10 years. Self-reported health is a well-established predictor of morbidity and mortality, covering physical, mental and social aspects of health [14, 15]. This research examines potential health differences by occupation in the total male and female working population. We further explore if and to what extent these results differ by age, activity status and socio-economic position.

## Method

Data were derived from an anonymous record linkage between the Belgian censuses of 1991 and 2001. Statistics Belgium performed the linkage at the individual level using unique identification numbers for each citizen. An additional linkage with the population register was performed to account for migrations or deaths between the census dates [16]. The result is a rich, exhaustive dataset combining cross-sectional data at the time of the 1991 and 2001 census. The total Belgian working population aged 25 to 55 years was selected from the 1991 census and followed up until the 2001 census. A total of 1.7 million men and 1.1 million women were employed on 1 March 1991. In the period between the two censuses, 3.1% of male workers and 1.5% of female workers died. An overview of the number of deaths per occupation can be found in Table 3 in Appendix. Loss to follow-up due to emigration was 2.1% and 1.4% in the male and female working population, respectively. As a result, analyses are based on data from 1.5 million men and 1.0 million women who were at work on 1 March 1991 and resided in Belgium on 1 October 2001.

Health information was derived from the 2001 census using the question ‘How is your health in general?’ Self-reported health was dichotomized into good (very good/good coded 0) and poor (fair/bad/very bad coded 1) health. Health questions were not included in the 1991 census.

Occupational groups were composed using the 2-digit codes from the International Standard Classification of Occupations (ISCO-88) as recorded in the 1991 census. The ISCO codes discern skill levels and skill specialisation, referring to the level of complexity and the type of knowledge, tools and equipment used, respectively [17]. Persons working in sheltered workshops were not included because of the targeted health selection in specific industries, corresponding to 7368 disabled men and 4945 disabled women. Both among men and women, the largest occupational group is ‘office clerks’ with respectively 12% of working men and 23% of working women. Other important occupational groups for men include ‘extraction and building trades’ and ‘metal, machinery and related trades’, with both employing approximately 9% of working men. Among women, we find a substantial number of women working in elementary services (11%) and in professional teaching jobs (11%).

Tables 4 and 5 in Appendix provide a comprehensive overview of the classification for men and women. Detailed occupational information is not available for 2001. The dataset does include information on the activity status in 2001. Respondents were asked to which category of persons they belong. Possible answers included students, actively employed persons, first-time jobseekers, other unemployed and (early) retirees. We used this information to determine who is still active, unemployed, (pre)retired or inactive due to personal, health or familial reasons.

Logistic regression analyses were performed for poor self-reported health by occupational group. Analyses were performed for men and women separately due to well-established sex differences in the distribution of risk factors for poor self-reported health [18]. Odds Ratios (OR) and 95% Confidence Intervals (CI) were computed with the sex-specific population that is still actively employed in 2001 as reference population. The majority of the active population in 1991 was still employed ten years later with 75% of men and 70% of women. These groups represent the healthiest individuals and provide an insight in the “acceptable” health situation to remain actively employed.

Analyses were performed using STATA/MP version 13.1. Three control variables were added step-wise. First, all models controlled for age measured continuous in years at the time of the 2001 census. Age is an important factor as health deteriorates as people grow older [19].

Second, activity status in 2001 was also included as a control to investigate the associations between poor self-reported health and the transition into the non-active population. Several mechanisms can play a role in the association between activity status and health [20]. Workers may leave employment because of a work-related disease. In the case of a non-occupational disease, workers may also have to leave employment because working conditions have become too strenuous or because of the gravity of the condition.

Third, socio-economic background was examined. Multiple studies have reported an association between poor health and low socio-economic position (SEP) [21–23]. Occupation is an important component of SEP which is a composite measure for an individual’s place in the social structure [24]. Because the workplace is an important source of social determinants of health, work is potentially closely related to various other material and social indicators [25]. Careful consideration of the socio-economic background is warranted when investigating occupational health differences [26]. Information on housing and ownership was used for this purpose. Housing conditions may have direct health effects [27]. In addition, housing status has also been reported to be a good indicator for material circumstances as it entails past (e.g. inheritance), present (e.g. wage) and future (e.g. mortgage) income perspectives [28]. The variable combines information on housing comfort and home ownership at the time of the 2001 census. Tenants and home owners with low, medium and high housing comfort were distinguished. Homes with low comfort require large repairs. Mid- and high-quality homes have central heating and are > 35 m^2^ and > 85 m^2^, respectively.

## Results

Table 1 presents the number and share of persons in poor health among the 1991 Belgian workforce with a 10-year lag. Generally, two out of ten workers reported their health to be poor ten years later (men 23%; women 21%). For men, the highest percentage was found among services elementary occupations (31%) and extraction and building trades workers (31%). The mean age for both occupational groups was 48 years in 2001, which is slightly lower than the total male average. For women, services elementary occupations had the highest share for reporting poor health (32%). This occupational group is a little older than average with a mean age of 49 in 2001.

**Table 1:** Poor self-reported health as reported in the 2001 Belgian census by occupational group at the time of the 1991 Belgian census

Occupational group in 1991 (ISCO code)	Men				Women			
	N 2001	Age	SRH	SRH%	N 2001	Age	SRH	SRH%
Legislators and senior officials (11)	4291	53	703	16%	1213	51	201	17%
Corporate managers (12)	129,042	51	20,476	16%	39,553	49	6949	18%
Managers of small enterprises (13)	58,998	50	14,422	24%	37,197	50	9776	26%
Physical, math. and engin. science professionals (21)	43,288	47	4721	11%	6691	42	582	9%
Life science and health professionals (22)	28,794	48	3264	11%	71,199	46	9811	14%
Teaching professionals (23)	65,059	51	12,410	19%	109,756	49	19,031	17%
Other professionals (24)	54,424	49	8736	16%	40,270	47	6137	15%
Physical and engin. science assoc. professionals (31)	114,747	50	23,736	21%	18,953	47	3243	17%
Life science and health assoc. professionals (32)	10,238	48	1358	13%	20,738	46	2832	14%
Teaching associate professionals (33)	7924	48	1459	18%	15,560	47	2915	19%
Other associate professionals (34)	58,711	49	10,419	18%	37,696	48	5995	16%
Office clerks (41)	182,752	49	37,729	21%	233,708	47	41,732	18%
Customer services clerks (42)	6389	48	1247	20%	24,206	47	5233	22%
Personal and protective services workers (51)	58,194	48	13,694	24%	83,322	47	21,686	26%
Salespersons and demonstrators (52)	22,889	47	4686	20%	65,966	47	14,299	22%
Skilled agricultural and related workers (61)	37,704	50	9499	25%	13,147	52	3379	26%
Extraction and building trades workers (71)	134,765	48	41,308	31%	1622	48	470	29%
Metal, machinery and related trades workers (72)	131,878	48	34,250	26%	11,241	47	2973	26%
Precision, (handi-)craft and related trades workers (73)	20,021	49	5059	25%	4509	46	1013	22%
Other craft and related trades workers (74)	43,324	48	11,153	26%	28,683	47	7128	25%
Stationary plant and related operators (81)	23,014	48	6014	26%	2010	48	573	29%
Machine operators and assemblers (82)	40,326	48	10,489	26%	28,904	46	7445	26%
Drivers and mobile plant operators (83)	98,848	49	28,029	28%	2586	47	706	27%
Services elementary occupations (91)	60,491	49	18,779	31%	114,190	49	37,012	32%
Agricultural and related labourers (92)	431	43	96	22%	2136	53	637	30%
Labourers in mining, constr., manuf. & transport (93)	79,761	48	22,627	28%	13,648	47	3889	28%
Armed forces (100)	24,288	46	4344	18%	1890	44	344	18%
Total	1,540,591	49	350,707	23%	1,030,594	48	215,991	21%

Abbreviations: *N2001* Study population census 2001, *Age* Mean age in 2001 in years, *SRH* absolute number of persons reporting poor health in 2001, *SRH%* percentage of persons reporting poor health from the 2001 population

The occupational groups with the fewest workers reporting poor health in 2001 were scientific professions. Approximately 10% of workers in physical, mathematical and engineering science reported poor health (men 11%; women 9%). We found a similar result among life science and health professionals (men 11%; women 14%).

**Fig. 1:**
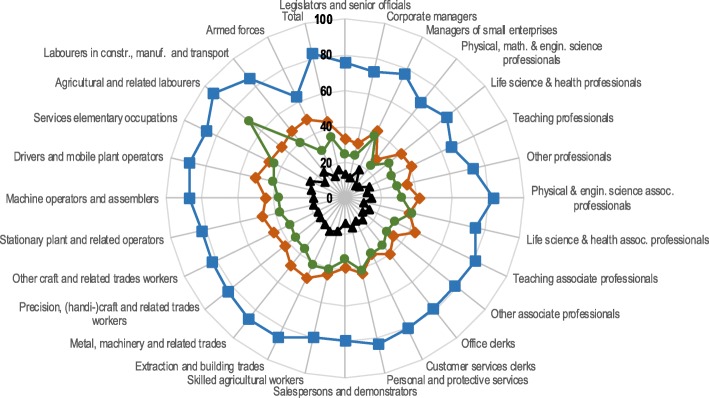
Percentage of men reporting poor health by occupational group in the 1991 Belgian census and activity status in the 2001 Belgian census. *Legend:*

**Fig. 2:**
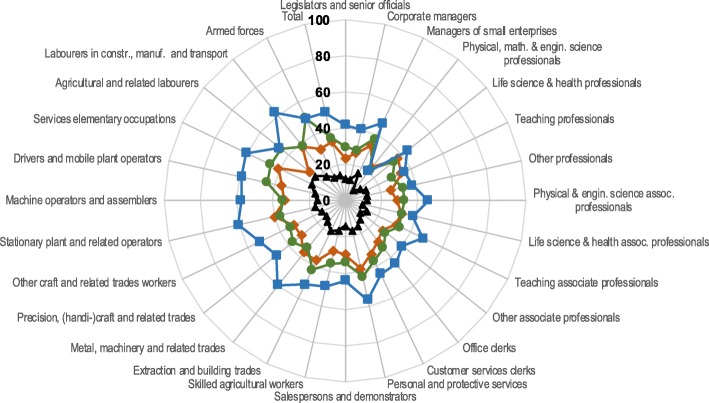
Percentage of women reporting poor health by occupational group in the 1991 Belgian census and activity status in the 2001 Belgian census. *Legend:*

Figures 1 and 2 present the share of persons in poor health per occupational group in 1991 and by activity status in 2001. The results for men show a clear gradient by activity status. Percentages were highest among those who left employment because of personal reasons with results ranging from 63% for armed forces and 94% for agricultural labourers. Unemployed men had a higher relative share for poor health than retired men. One exception to the pattern was found among agricultural labourers, where retired workers (68%) had a higher share to report poor health than unemployed workers (45%). Workers that were still active had the lowest percentages from 9% among physical, mathematical and engineering professionals to 22% among service elementary workers.

The pattern for women was more condensed than for men, meaning the shares by activity status do not differ as much among women as among men. This is mostly because of the relatively low share of women to report poor health after they left employment due to personal reasons. Percentages for this group ranged between 21% for physical, mathematical and engineering professionals and 62% for labourers in construction, manufacturing and transport. Results for unemployed women were highly similar to the findings for retirees. Again, active workers had the lowest share to report poor health with 7% of physical, mathematical and engineering professionals and 21% of agricultural labourers.

Table 2 presents ORs for poor self-reported health by sex. Predictor variables in model1 are respondents’ 1991 occupational group and their age at the time of the 2001 census. Model 2 adds the activity status in 2001 and model 3 finally adds the housing status in 2001.

**Table 2:** Results of multivariate logistic regression models predicting 2001 self-reported poor health by sex in Belgium, Odds Ratios and 95% Confidence Intervals, sorted on ORs for men in model 1

	Men						Women					
	Model 1		Model 2		Model 3		Model 1	Model 2		Model 3		
	OR	CI	OR	CI	OR	CI	OR	CI	OR	CI	OR	CI
Occupational group 1991 (Active pop. 1991 & 2001 = Ref)	1.00	–	1.00	–	1.00	–	1.00	–	1.00		1.00	
Extraction and building trades workers	2.08	(2.05–2.10)	1.42	(1.40–1.44)	1.36	(1.34–1.38)	2.15	(1.93–2.40)	1.34	(1.19–1.50)	1.27	(1.13–1.43)
Services elementary occupations	2.06	(2.03–2.10)	1.47	(1.44–1.50)	1.35	(1.33–1.38)	2.37	(2.34–2.41)	1.55	(1.52–1.57)	1.41	(1.39–1.43)
Labourers in mining, constr., manuf. and transport	1.90	(1.86–1.93)	1.34	(1.32–1.36)	1.23	(1.21–1.25)	2.21	(2.12–2.29)	1.40	(1.34–1.46)	1.29	(1.24–1.34)
Drivers and mobile plant operators	1.81	(1.79–1.84)	1.32	(1.30–1.35)	1.23	(1.21–1.25)	2.04	(1.87–2.23)	1.34	(1.22–1.47)	1.25	(1.14–1.37)
Agricultural and related labourers	1.79	(1.42–2.26)	1.36	(1.06–1.74)	1.25	(0.97–1.60)	1.60	(1.45–1.76)	1.13	(1.03–1.25)	1.09	(0.99–1.21)
Machine operators and assemblers	1.71	(1.67–1.75)	1.24	(1.21–1.27)	1.17	(1.14–1.20)	2.02	(1.96–2.07)	1.26	(1.23–1.30)	1.19	(1.15–1.22)
Metal, machinery and related trades workers	1.66	(1.64–1.69)	1.24	(1.22–1.25)	1.19	(1.18–1.21)	1.94	(1.86–2.03)	1.23	(1.18–1.29)	1.18	(1.13–1.24)
Stationary plant and related operators	1.64	(1.59–1.69)	1.23	(1.19–1.27)	1.18	(1.14–1.21)	2.15	(1.94–2.37)	1.38	(1.24–1.53)	1.30	(1.17–1.45)
Other craft and related trades workers	1.61	(1.57–1.64)	1.07	(1.05–1.10)	1.02	(0.99–1.04)	1.79	(1.75–1.85)	1.08	(1.05–1.11)	1.04	(1.01–1.07)
Precision, (handi-)craft and related trades workers	1.49	(1.44–1.54)	1.08	(1.04–1.12)	1.04	(1.01–1.08)	1.66	(1.54–1.78)	1.05	(0.98–1.14)	1.01	(0.94–1.09)
Personal and protective services workers	1.44	(1.42–1.47)	1.15	(1.13–1.18)	1.11	(1.09–1.13)	1.91	(1.88–1.95)	1.34	(1.32–1.37)	1.27	(1.25–1.30)
Skilled agricultural and related workers	1.41	(1.37–1.44)	1.08	(1.05–1.11)	1.02	(0.99–1.04)	1.39	(1.34–1.45)	0.94	(0.90–0.98)	0.91	(0.87–0.94)
Managers of small enterprises	1.33	(1.30–1.35)	0.96	(0.94–0.98)	0.95	(0.93–0.98)	1.60	(1.56–1.64)	1.00	(0.98–1.03)	0.95	(0.93–0.98)
Salespersons and demonstrators	1.25	(1.21–1.29)	0.91	(0.88–0.94)	0.90	(0.86–0.93)	1.51	(1.48–1.54)	0.98	(0.96–1.00)	0.95	(0.93–0.97)
Office clerks	1.15	(1.13–1.16)	0.95	(0.94–0.96)	0.96	(0.95–0.98)	1.19	(1.18–1.21)	0.92	(0.91–0.93)	0.93	(0.92–0.94)
Customer services clerks	1.13	(1.06–1.20)	0.91	(0.85–0.97)	0.92	(0.86–0.99)	1.54	(1.49–1.59)	1.13	(1.09–1.16)	1.11	(1.07–1.15)
Armed forces	1.12	(1.08–1.16)	0.95	(0.91–0.98)	0.93	(0.90–0.97)	1.57	(1.40–1.77)	1.29	(1.15–1.46)	1.23	(1.09–1.39)
Physical and engineering science assoc. professionals	1.11	(1.09–1.13)	0.91	(0.90–0.92)	0.93	(0.91–0.94)	1.12	(1.08–1.17)	0.86	(0.83–0.90)	0.88	(0.84–0.91)
Teaching associate professionals	1.07	(1.01–1.13)	0.93	(0.87–0.98)	0.96	(0.90–1.02)	1.26	(1.21–1.32)	1.00	(0.96–1.05)	1.02	(0.97–1.06)
Other associate professionals	0.93	(0.91–0.95)	0.74	(0.72–0.76)	0.76	(0.75–0.78)	0.98	(0.95–1.01)	0.72	(0.70–0.74)	0.74	(0.72–0.76)
Teaching professionals	0.90	(0.88–0.92)	0.81	(0.80–0.83)	0.89	(0.87–0.91)	0.99	(0.97–1.01)	0.85	(0.83–0.86)	0.92	(0.91–0.94)
Other professionals	0.83	(0.81–0.85)	0.72	(0.70–0.74)	0.77	(0.75–0.78)	0.99	(0.96–1.02)	0.82	(0.79–0.84)	0.86	(0.83–0.88)
Corporate managers	0.74	(0.73–0.75)	0.60	(0.59–0.61)	0.65	(0.64–0.66)	1.00	(0.97–1.03)	0.73	(0.70–0.75)	0.74	(0.72–0.76)
Life science and health assoc. professionals	0.72	(0.68–0.77)	0.63	(0.59–0.67)	0.68	(0.64–0.72)	0.90	(0.87–0.94)	0.73	(0.70–0.76)	0.76	(0.73–0.80)
Legislators and senior officials	0.68	(0.62–0.73)	0.62	(0.57–0.67)	0.68	(0.63–0.74)	0.83	(0.71–0.96)	0.70	(0.60–0.82)	0.75	(0.64–0.87)
Physical, math. and engineering science professionals	0.61	(0.59–0.63)	0.54	(0.53–0.56)	0.60	(0.58–0.62)	0.71	(0.65–0.77)	0.58	(0.53–0.64)	0.63	(0.58–0.69)
Life science and health professionals	0.59	(0.57–0.61)	0.54	(0.52–0.56)	0.61	(0.59–0.63)	0.95	(0.92–0.97)	0.79	(0.77–0.81)	0.83	(0.81–0.85)
Intercept	0.01	(0.01–0.01)	0.02	(0.02–0.02)	0.01	(0.01–0.01)	0.01	(0.01–0.01)	0.01	(0.01–0.01)	0.01	(0.01–0.01)
Age	1.06	(1.06–1.06)	1.05	(1.05–1.05)	1.05	(1.05–1.05)	1.07	(1.07–1.07)	1.07	(1.06–1.06)	1.06	(1.06–1.06)
Activity status (Employed = Ref)			1.00	–	1.00	–			1.00		1.00	
Unemployed			3.61	(3.61–3.75)	3.29	(3.22–3.35)			2.69	(2.64–2.74)	2.58	(2.54–2.63)
Retired			1.35	(1.35–1.39)	1.33	(1.32–1.35)			1.43	(1.41–1.46)	1.42	(1.40–1.45)
Personal reasons			17.38	(17.38–18.17)	16.76	(16.40–17.14)			4.84	(4.77–4.92)	4.90	(4.82–4.98)
Other			3.53	(3.53–3.76)	3.30	(3.20–3.41)			2.57	(2.49–2.65)	2.50	(2.42–2.58)
Missing			2.64	(2.64–2.95)	2.38	(2.25–2.52)			1.91	(1.80–2.03)	1.79	(1.68–1.9)
Housing status (Own –high quality = Ref)					1.00	–					1.00	
Own -medium quality					1.32	(1.31–1.33)					1.29	(1.27–1.3)
Own- low quality					1.55	(1.54–1.56)					1.47	(1.46–1.49)
Rent -high quality					1.25	(1.23–1.27)					1.31	(1.28–1.33)
Rent -medium quality					1.64	(1.62–1.66)					1.75	(1.72–1.78)
Rent - low quality					2.03	(2.01–2.06)					2.13	(2.10–2.16)
Missing					1.90	(1.88–1.93)					1.79	(1.76–1.82)
Model evaluation												
Degrees of freedom	28		33		39		28		33		39	
Likelihood ratio test	136,604.33***		243,474.39***		267,517.61***		94,204.91***		138,590.9***		152,124.72***	
Pseudo R^2^	0.05		0.09		0.10		0.06		0.08		0.09	

*** *p* ≤ 0.001

Occupational variation in ORs for poor self-reported health was similar for men and women. Compared to active workers in both 1991 and 2001, working in services elementary occupations, craft and construction was associated with an increased likelihood to report poor health. The three occupations with the highest age-adjusted ORs were extraction and building trade workers (OR_male_ 2.08 CI 2.05–2.10; OR_female_ 2.15 CI 1.93–2.40); services elementary workers (OR_male_ 2.06 CI 2.03–2.10; OR_female_ 2.37 CI 2.34–2.41); and labourers in mining, construction, manufacturing and transport (OR_male_ 1.90 CI 1.86–1.93; OR_female_ 2.21 CI 2.12–2.29). Men and women in teaching, scientific, health-related and managerial positions had lower age-adjusted ORs for poor self-reported health. Among women, the lowest ORs were found among physical, mathematical and engineering science professionals with 0.71 (CI 0.65–0.77). Their male colleagues had a similar OR (0.61 CI 0.59–0.63), but the lowest OR among men was found among life science and health professionals with 0.59 (CI 0.57–0.61).

The pattern in health differences by occupational group remained clear after controlling for activity status and housing status in 2001.The inclusion of activity status caused ORs in model 2 to decrease considerably for all occupational groups. Compared to workers that were still active in 2001, non-active statuses consistently had higher ORs for poor self-reported health. Leaving employment because of health, familial or other personal reasons was associated with the highest ORs for poor health. Generally, unemployed men and women had higher ORs than retirees with reference to the active population. ORs for occupations converged slightly in model 3 after adding housing status to the model. People living in low-quality housing were more likely to report poor health than those who live in high-quality housing. Home owners had lower ORs than tenants.

## Discussion

This study investigated census-linked data to examine differences in health by occupation among the total Belgian workforce in 1991. The main aim of this research was to provide an overview of self-reported health status in 2001 among 27 occupational groups with a 10-year lag. We found large health inequalities in the Belgian workforce for both sexes, especially in lower qualified occupations. Our results indicate that workers in physically demanding jobs had an increased likelihood to report poor health with reference to the active population in 1991 and 2001. Considerable variation existed within manual jobs as age-adjusted ORs ranged between approximately 1.40 and 2.00. Even higher results were observed among women, where age-adjusted ORs were up to 2.37 (CI 2.34–2.41) for services elementary occupations. Teaching, health-related and managerial jobs were associated with lower ORs for poor self-reported health. The pattern in occupational health differences remained the same after controlling for activity status and socio-economic status.

Occupation, age, activity status and housing status explained up to 10% and 9% of health differences among men and women, respectively. The largest contribution in explained variance was found after controlling for activity status. Especially ORs among manual workers experienced a stark decline after taking activity status into account. A potential explanation lies in differential healthy worker effects by occupational group [29]. Health problems in late middle age may create a discrepancy between the individual capabilities and the job requirements. Because of the physically demanding nature of manual labour, these workers may be more likely to encounter a mismatch than non-manual workers. In addition, the accumulation of unfavourable working conditions in these occupations has been reported to affect workers’ health [30]. As a result, more manual workers may quit their jobs due to health reasons compared to other workers, creating larger health differences by activity status in manual occupations.

Housing status did not seem to explain much of the differences in self-reported health by occupation. The inclusion of housing status modified ORs only slightly. The largest changes in ORs were among occupational groups with a specific socio-economic profile, such as services elementary occupations with a high proportion of cleaners.

The observed occupational health differences can perhaps be further explained by specific psychosocial or physical working conditions. Schütte and colleagues found that poor self-reported health is associated with hazardous working conditions, as well as with high psychological demands, low rewards and work-life imbalance for both men and women [18]. Several other studies have reported an influence of high job demands, job insecurity and repetitive work on occupational health differences [31–33]. Future research is needed to understand which work-related health risks are of importance for the investigated occupational groups.

The key strength of this study is the availability of a large, exhaustive dataset, which allowed us to study the total 1991 Belgian workforce. High-quality information was drawn from comprehensive and reliable national data sources [34]. The findings for 27 specific occupational groups complement existing international research based on fewer and broader categories. As a result, the findings provide a nuanced overview of occupational health in Belgium for the first time. In addition, this research adds to the growing body of literature on work-related health differences among women. Earlier studies report mixed and contradictory results [4, 6, 19, 35–37]. In accordance with recent French findings [6], we found relatively large occupational differences in women’s health. The pattern in self-reported health by occupation is clearly similar for men and women although results cannot be directly compared because of the use of sex-specific reference populations. We observed a difference in self-reported health by activity status between men and women. Men who left employment because of personal, health or familial reasons have markedly higher ORs than women with reference to their respective reference populations. It is highly likely that poor health was the decisive factor for these men in leaving employment. Women in this age group may be more prone to stop working because of familial obligations such as caregiving or childrearing.

The study also has some limitations. Firstly, health was measured using a general self-reported health question. Answers reflect multiple health dimensions and are highly subject to individual perceptions, as well as to the wider socio-temporal context [38]. This entails results should be interpreted with consideration for cross-cultural differences. A common methodological issue with self-reported health is that health experiences affect the response rate. People in poor health may not be fit enough to answer the questions. Self-reported health from the 2001 Belgian census has been compared to the national health interview survey. Lorant and colleagues found fewer non-response and better representation of low socio-economic groups in the -mandatory- census [34].

Secondly, the repeated cross-sectional design does not capture the dynamic mechanisms underlying this complex relation between health and occupation. Previous research has found important effects of time-varying indicators [39, 40]. In this study, specific occupational information is only available for one point in time. Job changes may have occurred over the period of 10 year, which may alter our results slightly. In our opinion, transfers from one occupational group to another in 1991 will be scarce given the use of relatively broad categories of occupations. Health selection in and out of employment has been reported to be more important than changes between jobs [41]. By controlling for activity status in 2001, we have accounted for possible healthy worker effects due to inactivity. However, it is possible that those experiencing very poor health, may have died or emigrated in the 10-year lag period. As a result of this potential underestimation in the worst-off professions, health inequalities between occupational groups may be even larger than presented in this study.

Thirdly, potential effects from part-time and full-time work are not investigated in the present study. An association between poor health and part-time employment has been reported in previous research [42]. It is possible that our results for women may alter somewhat when considering differences in work time. According to the 1991 census, 64% of working women were employed full-time with proportions ranging from 45% among customers service clerks to 98% in the armed forces. In contrast, the overall majority (95%) of active Belgian men worked full-time in 1991 with little variation across occupations. This topic should be investigated in future research with consideration of the complexities of contextual gender differences (e.g. child rearing tasks, relationship status, different working conditions and welfare state provisions) [43, 44].

Fourthly, the lack of suitable occupational data is a major obstacle for Belgian longitudinal analyses on this topic. The results for the 1991 working population may not reflect current-day differences in health situation by occupation. Although the occupational groups under investigation are still highly relevant, the Belgian workforce has undergone some important socio-demographic and economic changes over the last decades, as most West-European countries [45]. The presented findings are based on the most recent data available for a nationwide analysis of health differences by occupation in Belgium.

This research quantifies an important policy challenge in Belgium. This study shows a continuum of health risks with a clear hierarchy by occupation even after a 10-year lag time. Policy makers should invest in reducing health disparities by occupation. We stress the importance of additional research and policy efforts targeting manual labour jobs.

We also call policymakers’ attention to the large health differences by occupation in the female working population, especially considering the increased labour market participation of women during the last decades. According to data from the International Labour Organization, the overall female labour force participation increased from 38% in 1992 to 48% in 2017 [46].

## Conclusion

This study provided an overview of health differences among 27 types of Belgian workers. Both male and female workers in physically demanding occupations were more likely to report poor health. Significantly fewer workers in teaching, health-related and managerial jobs reported poor health. Large differences were observed between activity statuses -particularly in men- as found in previous research [20]. The current study confirms earlier findings of a negative association between socio-economic position and poor self-reported health [21–23]. To our knowledge, this is the first Belgian study to provide insights in the health situation by occupation with a ten year-lag. For now, we can only speculate on which health problems are at the root based on these results. Future research is required to determine the underlying mechanisms of the presented occupational health differences.

## Appendix

**Table 3:** Population in the 1991 and 2001 Belgian censuses with mortality information in the intermediate period from the National Register

Occupational group in 1991 (ISCO code)	Men				Women			
	N 1991	D9101	D%	N 2001	N 1991	D 9101	D%	N 2001
Legislators & senior officials (11)	4342	109	3%	4291	1344	23	2%	1213
Corporate managers (12)	145,563	4055	3%	129,042	43,608	674	2%	39,553
Managers of small enterprises (13)	67,433	2508	4%	58,998	41,011	915	2%	37,197
Physical, math. & engin. Science professionals (21)	47,276	804	2%	43,288	7200	56	1%	6691
Life science & health professionals (22)	31,203	666	2%	28,794	75,324	933	1%	71,199
Teaching professionals (23)	69,505	1812	3%	65,059	115,784	1568	1%	109,756
Other professionals (24)	60,675	1623	3%	54,424	43,739	609	1%	40,270
Physical & engin. Science assoc. professionals (31)	123,646	3406	3%	114,747	20,123	249	1%	18,953
Life science & health assoc. professionals (32)	11,017	260	2%	10,238	21,965	268	1%	20,738
Teaching associate professionals (33)	8482	212	2%	7924	16,454	235	1%	15,560
Other associate professionals (34)	64,442	1915	3%	58,711	40,401	540	1%	37,696
Office clerks (41)	198,520	6333	3%	182,752	248,577	3460	1%	233,708
Customer services clerks (42)	6891	206	3%	6389	25,602	322	1%	24,206
Personal & protective services workers (51)	64,538	2301	4%	58,194	89,339	1249	1%	83,322
Salespersons & demonstrators (52)	25,304	748	3%	22,889	70,406	931	1%	65,966
Skilled agricultural & related workers (61)	41,208	1394	3%	37,704	14,030	228	2%	13,147
Extraction & building trades workers (71)	148,321	5072	3%	134,765	1766	32	2%	1622
Metal, machinery & related trades workers (72)	142,657	4209	3%	131,878	11,854	157	1%	11,241
Precision, (handi-)craft & related trades workers (73)	21,738	695	3%	20,021	4782	58	1%	4509
Other craft & related trades workers (74)	47,410	1484	3%	43,324	30,334	370	1%	28,683
Stationary plant &related operators (81)	24,926	806	3%	23,014	2138	25	1%	2010
Machine operators & assemblers (82)	43,824	1294	3%	40,326	30,562	376	1%	28,904
Drivers & mobile plant operators (83)	109,143	4130	4%	98,848	2784	50	2%	2586
Services elementary occupations (91)	67,620	2749	4%	60,491	122,441	2085	2%	114,190
Agricultural & related labourers (92)	459	12	3%	431	2311	59	3%	2136
Labourers in mining, constr., manuf. & transport (93)	87,529	3226	4%	79,761	14,503	179	1%	13,648
Armed forces (100)	26,704	713	3%	24,288	2004	26	1%	1890
Total	1,690,376	52,742	3%	1,540,591	1,100,386	15,677	1%	1,030,594

**Table 4:** Occupational categories in English, Dutch and French [47]

ISCO	English	Dutch	French
11	Legislators, senior officials and managers	Leden van de wetgevende en uitvoerende macht en hogere kaderleden van het openbaar bestuur	Membres de l’exécutif et des corps législatifs, et cadres supérieurs de l’administration publique
12	Corporate managers	Bedrijfsleider en hoger kaderpersoneel	Directeurs de société
13	Managers of small entertprises	Directeurs en beheerders van kleine ondernemingen	Dirigeants et gérants
21	Physical, mathematical and engineering science professionals	Specialisten in de fysische, wiskundige en technische wetenschappen	Spécialistes des sciences physiques, mathématiques et techniques
22	Life science and health professionals	Specialisten in de medische en biowetenschappen	Spécialistes des sciences de la vie et de la santé
23	Teaching professionals	Specialisten in het onderwijs	Spécialistes de l’enseignement
24	Other professionals	Andere specialisten in intellectuele en wetenschappelijke beroepen	Autres spécialistes des professions intellectuelles et scientifiques
31	Physical and engineering science associate professionals	Ondergeschikt personeel in de fysische, wiskundige en technische wetenschappen	Professions intermédiaires des sciences physiques et techniques
32	Life science an dhealth associate professionals	Ondergeschikt personeel in de medische wetenschappen	Professions intermédiaires des sciences de la vie et de la santé
33	Teaching associate professionals	Ondergeschikt personeel in het onderwijs	Professions intermédiaires de l’enseignement
34	Other associate professionals	Ander ondergeschikt personeel in de intellectuele en wetenschappelijke beroepen	Autres professions intermédiaires
41	Office clerks	Kantoorbedienden	Employés de bureau
42	Customer servies clerks	Receptionisten, kassiers, loketbedienden en dergelijke	Employés de réception, caissiers, guichetiers et assimilés
51	Personal and protective services workers	Dienstverlenend en veiligheidspersoneel	Personnel des services directs aux particuliers et des services de protection et de sécurité
52	Models, salespersons and demonstrators	Modellen, verkopers en demonstrateurs	Modèles, vendeurs et démonstrateurs
61	Skilled argicultural and fishery workers	Voor de markt producerende landbouwers en geschoolde arbeiders in de landbouw en de visserij	Agriculteurs et ouvriers qualifiés de l’agriculture et de la pêche destinées aux marchés
71	Extraction and building trades workers	Ambachtslieden en vakarbeiders in de winning van delfstoffen en de bouwnijverheid	Artisans et ouvriers des métiers de l’extraction et du bâtiment
72	Metal,machinery and related trades workers	Ambachtslieden en vakarbeiders in de metallurgie, de metaalverwerkende nijverheid, de machinebouw en dergelijke	Artisans et ouvriers des métiers de la métallurgie, de la construction mécanique et assimilés
73	Precision, handicraft, craft printing and related trades workers	Ambachtslieden en vakarbeiders in de precisienijverheid, het kunstambacht, de drukkerijen en dergelijke	Artisans et ouvriers de la mécanique de précision, des métiers d’art, de l’imprimerie et assimilés
74	Other craft and related trades workers	Andere ambachtslieden en ambachtelijke vakarbeiders	Autres artisans et ouvriers des métiers de type artisanal
81	Stationary-plant and related operators	Fabrieksarbeiders aan vaste installaties en dergelijke	Conducteurs d’installations et de matériels fixes et assimilés
82	Machine operators and assemblers	Machine- en montage-arbeiders	Conducteurs de machines et ouvriers de l’assemblage
83	Drivers and mobile plant operators	Bestuurders van voertuigen, hijs-, hef- en transportwerktuigen	Conducteurs de véhicules et d’engins lourds de levage et de manœuvre
91	Sales and services elementary occupations	Ongeschoold dienstverlenend en verkoopspersoneel	Employés non qualifiés des services et de la vente
92	Agricultural, fishery and related labourers	Ongeschoolde arbeiders in de landbouw, visserij en dergelijke	Manœuvres de l’agriculture, de la pêche et assimilés
93	Labourers in mining, constructions, manufacturing and transport	Ongeschoolde arbeiders in de mijnbouw, de bouwnijverheid, de verwerkende nijverheid en het transport	Manœuvres des mines, du bâtiment et des travaux publics, des industries manufacturières et des transports
110	Armed forces	Strijdkrachten	Forces armées

**Table 5:** Study population by sex and occupation in the 1991 Belgian census

ISCO	Occupational group in 1991	M	M%	F	F%
11	Legislators and senior officials	4427	100.0	1269	100.0
1110	Legislators	129	2.9	59	4.6
1120	Senior government officials	3384	76.4	1043	82.2
1141	Senior officials of political-party organisations	12	0.3	11	0.9
1142	Senior officials of employers’, workers’ and other economic-interest organisations	380	8.6	74	5.8
1143	Senior officials of humanitarian and other special-interest organisations	170	3.8	82	6.5
110	Senior army officials	352	8.0	0	0.0
12	Corporate managers	134,047	100.0	41,217	100.0
1210	Directors and chief executives	57,924	43.2	14,808	35.9
1221	Production and operations department managers in agriculture, hunting, forestry and fishing	43	0.0	18	0.0
1224	Production and operations department managers in wholesale and retail trade	1425	1.1	704	1.7
1225	Production and operations department managers in restaurants and hotels	93	0.1	38	0.1
1229	Production and operations department managers not elsewhere classified	17,374	13.0	6713	16.3
1231	Finance and administration department managers	5951	4.4	752	1.8
1232	Personnel and industrial relations department managers	5108	3.8	1355	3.3
1233	Sales and marketing department managers	18,569	13.9	9097	22.1
1235	Supply and distribution department managers	162	0.1	24	0.1
1236	Computing services department managers	1199	0.9	153	0.4
1237	Research and development department managers	2069	1.5	1169	2.8
1239	Other department managers not elsewhere classified	24,130	18.0	6386	15.5
13	Managers of small enterprises	62,531	100.0	39,141	100.0
1311	General managers in agriculture, hunting, forestry and fishing	2521	4.0	652	1.7
1312	General managers in manufacturing	126	0.2	6	0.0
1313	General managers in construction	4124	6.6	55	0.1
1314	General managers in wholesale and retail trade	33,613	53.8	22,691	58.0
1315	General managers of restaurants and hotels	12,922	20.7	12,247	31.3
1317	General managers of business services	87	0.1	28	0.1
1318	General managers in personal care, cleaning and related services	395	0.6	869	2.2
1319	General managers not elsewhere classified	8743	14.0	2593	6.6
21	Physical, mathematical and engineering science professionals	44,553	100.0	6876	100.0
2111	Physicists and astronomers	79	0.2	43	0.6
2112	Meteorologists	18	0.0	3	0.0
2113	Chemists	274	0.6	104	1.5
2114	Geologists and geophysicists	117	0.3	27	0.4
2121	Mathematicians and related professionals	87	0.2	51	0.7
2122	Statisticians	50	0.1	28	0.4
2131	Computer systems designers and analysts	11,535	25.9	2071	30.1
2132	Computer programmers	7968	17.9	2206	32.1
2139	Computing professionals not elsewhere classified	1251	2.8	94	1.4
2141	Architects, town and traffic planners	5451	12.2	1384	20.1
2142	Civil engineers	1489	3.3	73	1.1
2143	Electrical engineers	454	1.0	7	0.1
2144	Electronics and telecommunications engineers	952	2.1	29	0.4
2145	Mechanical engineers	196	0.4	2	0.0
2146	Chemical engineers	397	0.9	78	1.1
2147	Mining engineers, metallurgists and related professionals	49	0.1	2	0.0
2148	Cartographers and surveyors	1204	2.7	77	1.1
2149	Architects, engineers and related professionals not elsewhere classified	12,982	29.1	597	8.7
22	Life science and health professionals	29,762	100.0	73,403	100.0
2211	Biologists, botanists, zoologists and related professionals	198	0.7	166	0.2
2212	Pharmacologists, pathologists and related professionals	95	0.3	58	0.1
2213	Agronomists and related professionals	411	1.4	72	0.1
2221	Medical doctors	14,166	47.6	4934	6.7
2222	Dentists	2988	10.0	1882	2.6
2223	Veterinarians	1674	5.6	422	0.6
2224	Pharmacists	2347	7.9	3510	4.8
2230	Nursing and midwifery professionals	7883	26.5	62,359	85.0
23	Teaching professionals	66,821	100.0	113,020	100.0
2310	College, university and higher education teaching professionals	4179	6.3	2064	1.8
2320	Secondary education teaching professionals	46,849	70.1	58,862	52.1
2331	Primary education teaching professionals	13,401	20.1	30,806	27.3
2332	Pre-primary education teaching professionals	402	0.6	18,886	16.7
2340	Special education teaching professionals	627	0.9	1171	1.0
2351	Education methods specialists	331	0.5	523	0.5
2352	School inspectors	217	0.3	68	0.1
2359	Other teaching professionals not elsewhere classified	815	1.2	640	0.6
24	Other professionals	56,647	100.0	41,848	100.0
2411	Accountants	15,150	26.7	9020	21.6
2412	Personnel and careers professionals	1344	2.4	603	1.4
2419	Business professionals not elsewhere classified	5728	10.1	2234	5.3
2421	Lawyers	5629	9.9	2909	7.0
2422	Judges	807	1.4	342	0.8
2429	Legal professionals not elsewhere classified	1913	3.4	827	2.0
2431	Archivists and curators	173	0.3	133	0.3
2432	Librarians and related information professionals	1027	1.8	2135	5.1
2441	Economists	222	0.4	83	0.2
2442	Sociologists, anthropologists and related professionals	134	0.2	129	0.3
2443	Philosophers, historians and political scientists	10	0.0	16	0.0
2444	Philologists, translators and interpreters	868	1.5	1062	2.5
2445	Psychologists	1091	1.9	1990	4.8
2446	Social work professionals	4350	7.7	11,339	27.1
2451	Authors, journalists and other writers	2938	5.2	1203	2.9
2452	Sculptors, painters and related artists	2990	5.3	2135	5.1
2453	Composers, musicians and singers	1049	1.9	367	0.9
2454	Choreographers and dancers	29	0.1	93	0.2
2455	Film, stage and related actors and directors	830	1.5	454	1.1
2460	Religious professionals	1419	2.5	74	0.2
2470	Public service administrative professionals	8946	15.8	4700	11.2
31	Physical and engineering science associate professionals	118,132	100.0	19,564	100.0
3111	Chemical and physical science technicians	1155	1.0	514	2.6
3112	Civil engineering technicians	929	0.8	44	0.2
3113	Electrical engineering technicians	8355	7.1	188	1.0
3114	Electronics and telecommunications engineering technicians	2596	2.2	93	0.5
3115	Mechanical engineering technicians	4763	4.0	128	0.7
3116	Chemical engineering technicians	8189	6.9	8881	45.4
3118	Draughtspersons	11,263	9.5	1401	7.2
3119	Physical and engineering science technicians not elsewhere classified	66,313	56.1	5414	27.7
3122	Computer equipment operators	1870	1.6	100	0.5
3131	Photographers and image and sound recording equipment operators	2308	2.0	647	3.3
3132	Broadcasting and telecommunications equipment operators	977	0.8	97	0.5
3133	Medical equipment operators	350	0.3	479	2.4
3141	Ships’ engineers	333	0.3	1	0.0
3142	Ships’ deck officers and pilots	1882	1.6	303	1.5
3143	Aircraft pilots and related associate professionals	600	0.5	31	0.2
3144	Air traffic controllers	536	0.5	80	0.4
3152	Safety, health and quality inspectors	5713	4.8	1163	5.9
32	Life science and health associate professionals	10,549	100.0	21,365	100.0
3211	Life science technicians	101	1.0	57	0.3
3212	Agronomy and forestry technicians	203	1.9	16	0.1
3213	Farming and forestry advisers	76	0.7	16	0.1
3221	Medical assistants	270	2.6	2300	10.8
3222	Sanitarians	53	0.5	42	0.2
3223	Dieticians and nutritionists	70	0.7	877	4.1
3224	Optometrists and opticians	1011	9.6	448	2.1
3225	Dental assistants	921	8.7	207	1.0
3226	Physiotherapists and related associate professionals	6261	59.4	9030	42.3
3228	Pharmaceutical assistants	569	5.4	3446	16.1
3229	Modern health associate professionals (except nursing) not elsewhere classified	359	3.4	2555	12.0
3231	Nursing associate professionals	634	6.0	2359	11.0
3242	Faith healers	21	0.2	12	0.1
33	Teaching associate professionals	8161	100.0	16,065	100.0
3310	Primary education teaching associate professionals	4146	50.8	9182	57.2
3320	Pre-primary education teaching associate professionals	17	0.2	1030	6.4
3330	Special education teaching associate professionals	2082	25.5	4940	30.8
3340	Other teaching associate professionals	1916	23.5	913	5.7
34	Other associate professionals	60,868	100.0	38,950	100.0
3411	Securities and finance dealers and brokers	416	0.7	133	0.3
3412	Insurance representatives	8283	13.6	2803	7.2
3413	Estate agents	471	0.8	198	0.5
3414	Travel consultants and organisers	861	1.4	537	1.4
3415	Technical and commercial sales representatives	26,570	43.7	5386	13.8
3416	Buyers	2747	4.5	1030	2.6
3417	Appraisers, valuers and auctioneers	96	0.2	29	0.1
3419	Finance and sales associate professionals not elsewhere classified	724	1.2	486	1.2
3421	Trade brokers	924	1.5	299	0.8
3422	Clearing and forwarding agents	209	0.3	42	0.1
3431	Administrative secretaries and related associate professionals	438	0.7	9516	24.4
3432	Legal and related business associate professionals	904	1.5	1449	3.7
3433	Bookkeepers	5186	8.5	12,751	32.7
3434	Statistical, mathematical and related associate professionals	109	0.2	152	0.4
3441	Customs and border inspectors	1270	2.1	381	1.0
3449	Customs, tax and related government associate professionals not elsewhere classified	2218	3.6	1269	3.3
3450	Police inspectors and detectives	7246	11.9	363	0.9
3471	Decorators and commercial designers	340	0.6	246	0.6
3472	Radio, television and other announcers	461	0.8	564	1.4
3474	Clowns, magicians, acrobats and related associate professionals	27	0.0	14	0.0
3475	Athletes, sportspersons and related associate professionals	1368	2.2	1302	3.3
41	Office clerks	188,232	100.0	240,961	100.0
4111	Stenographers and typists	5555	3.0	8392	3.5
4112	Word-processor and related operators	426	0.2	414	0.2
4113	Data entry operators	1990	1.1	3166	1.3
4114	Calculating-machine operators	205	0.1	463	0.2
4115	Secretaries	3513	1.9	40,178	16.7
4121	Accounting and bookkeeping clerks	3743	2.0	4213	1.7
4122	Statistical and finance clerks	21,858	11.6	19,280	8.0
4131	Stock clerks	8981	4.8	2823	1.2
4133	Transport clerks	2270	1.2	1084	0.4
4141	Library and filing clerks	196	0.1	602	0.2
4142	Mail carriers and sorting clerks	12,984	6.9	2525	1.0
4143	Coding, proof-reading and related clerks	354	0.2	521	0.2
4190	Other office clerks	126,157	67.0	157,300	65.3
42	Customer services clerks	6566	100.0	24,995	100.0
4211	Cashiers and ticket clerks	831	12.7	12,755	51.0
4212	Tellers and other counter clerks	3351	51.0	3754	15.0
4213	Bookmakers and croupiers	267	4.1	278	1.1
4214	Pawnbrokers and money-lenders	1	0.0	1	0.0
4215	Debt-collectors and related workers	262	4.0	200	0.8
4221	Travel agency and related clerks	549	8.4	1207	4.8
4222	Receptionists and information clerks	347	5.3	2125	8.5
4223	Telephone switchboard operators	958	14.6	4675	18.7
51	Personal and protective services workers	60,747	100.0	86,914	100.0
5111	Travel attendants and travel stewards	303	0.5	840	1.0
5112	Transport conductors	898	1.5	267	0.3
5113	Travel guides	89	0.1	165	0.2
5121	Housekeepers and related workers	1258	2.1	992	1.1
5122	Cooks	9895	16.3	20,174	23.2
5123	Waiters, waitresses and bartenders	5347	8.8	8952	10.3
5131	Child-care workers	21	0.0	3125	3.6
5132	Institution-based personal care workers	787	1.3	11,898	13.7
5133	Home-based personal care workers	301	0.5	21,359	24.6
5139	Personal care and related workers not elsewhere classified	80	0.1	336	0.4
5141	Hairdressers, barbers, beauticians and related workers	4648	7.7	14,014	16.1
5142	Companions and valets	186	0.3	2287	2.6
5143	Undertakers and embalmers	426	0.7	142	0.2
5149	Other personal services workers not elsewhere classified	13	0.0	36	0.0
5161	Fire-fighters	4605	7.6	20	0.0
5162	Police officers	16,712	27.5	861	1.0
5163	Prison guards	2067	3.4	148	0.2
5169	Protective services workers not elsewhere classified	13,111	21.6	1298	1.5
52	Salespersons and demonstrators	23,881	100.0	68,484	100.0
5210	Fashion and other models	121	0.5	1586	2.3
5220	Shop salespersons and demonstrators	21,790	91.2	65,100	95.1
5230	Stall and market salespersons	1970	8.2	1798	2.6
61	Skilled agricultural and related workers	39,462	100.0	13,726	100.0
6111	Field crop and vegetable growers	1231	3.1	722	5.3
6112	Tree and shrub crop growers	749	1.9	182	1.3
6113	Gardeners, horticultural and nursery growers	8460	21.4	1916	14.0
6114	Mixed-crop growers	24,185	61.3	9844	71.7
6121	Dairy and livestock producers	1503	3.8	600	4.4
6122	Poultry producers	585	1.5	270	2.0
6124	Mixed-animal producers	78	0.2	75	0.5
6141	Forestry workers and loggers	1881	4.8	41	0.3
6151	Aquatic-life cultivation workers	155	0.4	20	0.1
6153	Deep-sea fishery workers	628	1.6	56	0.4
71	Extraction and building trades workers	141,225	100.0	1700	100.0
7111	Miners and quarry workers	3354	2.4	72	4.2
7112	Shot firers and blasters	10	0.0	0	0.0
7113	Stone splitters, cutters and carvers	1344	1.0	30	1.8
7122	Bricklayers and stonemasons	27,467	19.4	112	6.6
7123	Concrete placers, concrete finishers and related workers	2442	1.7	17	1.0
7124	Carpenters and joiners	24,202	17.1	240	14.1
7129	Building frame and related trades workers not elsewhere classified	13,917	9.9	292	17.2
7131	Roofers	5029	3.6	25	1.5
7132	Floor layers and tile setters	4017	2.8	35	2.1
7133	Plasterers	3431	2.4	41	2.4
7134	Insulation workers	1526	1.1	22	1.3
7135	Glaziers	1160	0.8	93	5.5
7136	Plumbers and pipe fitters	14,795	10.5	106	6.2
7137	Building and related electricians	23,591	16.7	222	13.1
7141	Painters and related workers	13,106	9.3	337	19.8
7142	Varnishers and related painters	1363	1.0	49	2.9
7143	Building structure cleaners	471	0.3	7	0.4
72	Metal, machinery and related trades workers	136,747	100.0	11,611	100.0
7212	Welders and flamecutters	20,187	14.8	668	5.8
7213	Sheet-metal workers	6790	5.0	118	1.0
7214	Structural-metal preparers and erectors	61,271	44.8	8327	71.7
7215	Riggers and cable splicers	195	0.1	7	0.1
7216	Underwater workers	51	0.0	2	0.0
7221	Blacksmiths, hammer-smiths and forging-press workers	905	0.7	18	0.2
7222	Tool-makers and related workers	2803	2.0	118	1.0
7231	Motor vehicle mechanics and fitters	27,908	20.4	774	6.7
7232	Aircraft engine mechanics and fitters	615	0.4	8	0.1
7233	Agricultural- or industrial-machinery mechanics and fitters	7093	5.2	197	1.7
7241	Electrical mechanics and fitters	6415	4.7	1234	10.6
7242	Electronics fitters	1144	0.8	66	0.6
7243	Electronics mechanics and servicers	663	0.5	48	0.4
7244	Telegraph and telephone installers and servicers	332	0.2	11	0.1
7245	Electrical line installers, repairers and cable jointers	375	0.3	15	0.1
73	Precision, handicraft, craft printing and related trades workers	20,746	100.0	4665	100.0
7311	Precision-instrument makers and repairers	1089	5.2	171	3.7
7312	Musical instrument makers and tuners	82	0.4	3	0.1
7313	Jewellery and precious-metal workers	3567	17.2	1127	24.2
7321	Abrasive wheel formers, potters and related workers	1420	6.8	171	3.7
7322	Glass-makers, cutters, grinders and finishers	3625	17.5	447	9.6
7323	Glass engravers and etchers	52	0.3	20	0.4
7324	Glass, ceramics and related decorative painters	40	0.2	60	1.3
7331	Handicraft workers in wood and related materials	45	0.2	16	0.3
7332	Handicraft workers in textile, leather and related materials	406	2.0	60	1.3
7341	Compositors, typesetters and related workers	8179	39.4	1310	28.1
7342	Stereotypers and electrotypers	96	0.5	65	1.4
7343	Printing engravers and etchers	810	3.9	178	3.8
7344	Photographic and related workers	246	1.2	159	3.4
7345	Bookbinders and related workers	830	4.0	742	15.9
7346	Silk-screen, block and textile printers	259	1.2	136	2.9
74	Other craft and related trades workers	45,314	100.0	29,739	100.0
7411	Butchers, fishmongers and related food preparers	15,553	34.3	3825	12.9
7412	Bakers, pastry-cooks and confectionery makers	10,766	23.8	3587	12.1
7413	Dairy-products makers	61	0.1	20	0.1
7414	Fruit, vegetable and related preservers	9	0.0	19	0.1
7415	Food and beverage tasters and graders	8	0.0	0	0.0
7416	Tobacco preparers and tobacco products makers	268	0.6	1051	3.5
7421	Wood treaters	10	0.0	2	0.0
7422	Cabinet-makers and related workers	9955	22.0	1245	4.2
7423	Woodworking-machine setters and setter-operators	444	1.0	82	0.3
7424	Basketry weavers, brush makers and related workers	85	0.2	27	0.1
7431	Fibre preparers	315	0.7	147	0.5
7432	Weavers, knitters and related workers	3481	7.7	1328	4.5
7433	Tailors, dressmakers and hatters	489	1.1	6170	20.7
7434	Furriers and related workers	58	0.1	68	0.2
7435	Textile, leather and related pattern-makers and cutters	128	0.3	770	2.6
7436	Sewers, embroiderers and related workers	925	2.0	10,312	34.7
7437	Upholsterers and related workers	1216	2.7	287	1.0
7441	Pelt dressers, tanners and fellmongers	94	0.2	67	0.2
7442	Shoe-makers and related workers	1449	3.2	732	2.5
81	Stationary plant and related operators	23,831	100.0	2086	100.0
8111	Mining-plant operators	179	0.8	0	0.0
8112	Mineral-ore- and stone-processing-plant operators	168	0.7	0	0.0
8113	Well drillers and borers and related workers	453	1.9	4	0.2
8121	Ore and metal furnace operators	993	4.2	7	0.3
8122	Metal melters, casters and rolling-mill operators	4383	18.4	171	8.2
8123	Metal-heat-treating-plant operators	59	0.2	1	0.0
8124	Metal drawers and extruders	1698	7.1	329	15.8
8131	Glass and ceramics kiln and related machine operators	106	0.4	3	0.1
8141	Wood-processing-plant operators	1222	5.1	62	3.0
8142	Paper-pulp plant operators	15	0.1	3	0.1
8143	Papermaking-plant operators	3010	12.6	736	35.3
8151	Crushing-, grinding- and chemical-mixing machinery operators	480	2.0	11	0.5
8152	Chemical-heat-treating-plant operators	206	0.9	13	0.6
8153	Chemical-filtering- and separating-equipment operators	25	0.1	1	0.0
8154	Chemical-still and reactor operators (except petroleum and natural gas)	61	0.3	2	0.1
8155	Petroleum- and natural-gas-refining-plant operators	35	0.1	2	0.1
8159	Chemical-processing-plant operators not elsewhere classified	8313	34.9	493	23.6
8161	Power-production plant operators	947	4.0	102	4.9
8162	Steam-engine and boiler operators	669	2.8	31	1.5
8163	Incinerator, water-treatment and related plant operators	30	0.1	0	0.0
8171	Automated-assembly-line operators	706	3.0	100	4.8
8172	Industrial-robot operators	73	0.3	15	0.7
82	Machine operators and assemblers	41,942	100.0	29,982	100.0
8211	Machine-tool operators	7612	18.1	169	0.6
8212	Cement and other mineral products machine operators	362	0.9	5	0.0
8221	Pharmaceutical- and toiletry-products machine operators	974	2.3	855	2.9
8222	Ammunition- and explosive-products machine operators	15	0.0	11	0.0
8223	Metal finishing-, plating- and coating-machine operators	432	1.0	58	0.2
8224	Photographic-products machine operators	133	0.3	21	0.1
8229	Chemical-products machine operators not elsewhere classified	5811	13.9	1193	4.0
8231	Rubber-products machine operators	685	1.6	56	0.2
8232	Plastic-products machine operators	859	2.0	250	0.8
8240	Wood-products machine operators	307	0.7	35	0.1
8251	Printing-machine operators	3328	7.9	955	3.2
8252	Bookbinding-machine operators	1	0.0	0	0.0
8253	Paper-products machine operators	386	0.9	199	0.7
8261	Fibre-preparing-, spinning- and winding-machine operators	850	2.0	2563	8.5
8262	Weaving- and knitting-machine operators	708	1.7	475	1.6
8263	Sewing-machine operators	275	0.7	6707	22.4
8264	Bleaching-, dyeing- and cleaning-machine operators	1105	2.6	1661	5.5
8265	Fur- and leather-preparing-machine operators	124	0.3	21	0.1
8266	Shoemaking- and related machine operators	49	0.1	88	0.3
8271	Meat- and fish-processing-machine operators	30	0.1	9	0.0
8272	Dairy-products machine operators	1133	2.7	250	0.8
8273	Grain- and spice-milling-machine operators	410	1.0	25	0.1
8274	Baked-goods, cereal and chocolate-products machine operators	561	1.3	199	0.7
8275	Fruit-, vegetable- and nut-processing-machine operators	1764	4.2	174	0.6
8276	Sugar production machine operators	385	0.9	44	0.1
8277	Tea-, coffee-, and cocoa-processing-machine operators	184	0.4	19	0.1
8278	Brewers-, wine and other beverage machine operators	509	1.2	40	0.1
8279	Tobacco production machine operators	63	0.2	80	0.3
8281	Mechanical-machinery assemblers	3485	8.3	271	0.9
8282	Electrical-equipment assemblers	1655	3.9	868	2.9
8283	Electronic-equipment assemblers	516	1.2	126	0.4
8284	Metal-, rubber- and plastic-products assemblers	3187	7.6	688	2.3
8285	Wood and related products assemblers	6	0.0	1	0.0
8286	Paperboard, textile and related products assemblers	26	0.1	30	0.1
8290	Other machine operators and assemblers	4012	9.6	11,836	39.5
83	Drivers and mobile plant operators	103,341	100.0	2695	100.0
8311	Locomotive-engine drivers	4478	4.3	14	0.5
8312	Railway brakers, signallers and shunters	3167	3.1	52	1.9
8322	Car, taxi and van drivers	11,832	11.4	614	22.8
8323	Bus and tram drivers	9786	9.5	628	23.3
8324	Heavy truck and lorry drivers	49,177	47.6	781	29.0
8331	Motorised farm and forestry plant operators	65	0.1	1	0.0
8332	Earth-moving- and related plant operators	3924	3.8	30	1.1
8333	Crane, hoist and related plant operators	7882	7.6	30	1.1
8334	Lifting-truck operators	12,036	11.6	499	18.5
8340	Ships’ deck crews and related workers	994	1.0	46	1.7
91	Services elementary occupations	63,630	100.0	119,135	100.0
9112	Street vendors, non-food products	2905	4.6	1635	1.4
9120	Shoe cleaning and other street services elementary occupations	3	0.0	10	0.0
9131	Domestic helpers and cleaners	1043	1.6	51,649	43.4
9132	Helpers and cleaners in offices, hotels and other establishments	46,727	73.4	25,131	21.1
9133	Hand-launderers and pressers	577	0.9	4458	3.7
9141	Building caretakers	1392	2.2	2963	2.5
9142	Vehicle, window and related cleaners	3651	5.7	28,838	24.2
9151	Messengers, package and luggage porters and deliverers	40	0.1	14	0.0
9152	Doorkeepers, watchpersons and related workers	2349	3.7	4204	3.5
9153	Vending-machine money collectors, meter readers and related workers	438	0.7	94	0.1
9161	Garbage collectors	4210	6.6	134	0.1
9162	Sweepers and related labourers	295	0.5	5	0.0
92	Agricultural and related labourers	444	100.0	2242	100.0
9211	Farm-hands and labourers	439	98.9	2242	100.0
9212	Forestry labourers	5	1.1	0	0.0
93	Labourers in mining, construction, manufacturing and transport	83,206	100.0	14,189	100.0
9311	Mining and quarrying labourers	73	0.1	0	0.0
9312	Construction and maintenance labourers: roads, dams and similar constructions	11,058	13.3	89	0.6
9321	Assembling labourers	6243	7.5	5016	35.4
9322	Hand packers and other manufacturing labourers	8452	10.2	152	1.1
9333	Freight handlers	57,380	69.0	8932	63.0
110	Armed forces	25,228	100.0	1950	100.0

## Abbreviations

CI: 95% Confidence Intervals
OR: Odds Ratio
SEP: Socio-Economic Position
